# Effect of febuxostat on left ventricular diastolic function in patients with asymptomatic hyperuricemia: a sub analysis of the PRIZE Study

**DOI:** 10.1038/s41440-021-00752-9

**Published:** 2021-10-17

**Authors:** Kenya Kusunose, Hisako Yoshida, Atsushi Tanaka, Hiroki Teragawa, Yuichi Akasaki, Yoshihiro Fukumoto, Kazuo Eguchi, Haruo Kamiya, Kazuomi Kario, Hirotsugu Yamada, Masataka Sata, Koichi Node, The PRIZE Study Investigators, Koichi Node, Toyoaki Murohara, Teruo Inoue, Masataka Sata, Mitsuru Ohishi, Kotaro Yokote, Kazuomi Kario, Hirotaka Watada, Iichiro Shimomura, Munehide Matsuhisa, Yoshihiro Fukumoto, Koji Maemura, Yusuke Ohya, Yuichi Akasaki, Junya Ako, Hirohisa Amano, Kazutaka Aonuma, Yutaka Aoyama, Hirofumi Arai, Kuniya Asai, Machiko Asaka, Yoshifumi Awaji, Noriko Ban, Toshiaki Ban, Yasuko K. Bando, Hiroyuki Daida, Shunsuke Eguchi, Mami Enomoto, Yuichi Fujii, Akinori Fujikake, Masanori Fujimoto, Tomohiro Fujisaka, Shuichi Fujita, Satoki Fukae, Daiju Fukuda, Mieko Fukui, Yuhei Goriki, Shuichi Hamasaki, Tomoya Hara, Hiroshi Hasegawa, Kenichi Hashimoto, Mitsumasa Hata, Shiro Hata, Ryo Hayashida, Akihiro Higashi, Seiichiro Higuchi, Akihiro Honda, Satoshi Hoshide, Masaaki Hoshiga, Junko Hotchi, Sachiyo Igata, Yumi Ikehara, Youhei Inoue, Hiroko Ishigami, Masaharu Ishihara, Hideki Ishii, Tetsuya Ishikawa, Takashi Ishimatsu, Yusuke Ishiyama, Takahide Ito, Ayumi Ito, Toshiaki Kadokami, Haruo Kamiya, Soichiro Kashihara, Yoshihiro Kawamura, Kazuo Kitagawa, Yoshio Kobayashi, Satoshi Kodera, Seiji Koga, Hisashi Koide, Yuji Koide, Hiroshi Koiwaya, Hiroki Kojima, Eri Komai, Takaaki Komatsu, Shingo Kono, Takashi Kono, Yoshiaki Kubota, Akio Kuroda, Takanori Kuroyanagi, Akifumi Kushiyama, Kenya Kusunose, Tatsuya Maruhashi, Kazuo Matsunaga, Tomomi Matsuura, Takafumi Mayama, Daigo Mine, Masatoshi Miyamura, Ryota Morimoto, Hideaki Morita, Hidekazu Nagano, Hidemitsu Nakagawa, Katsunori Nakamura, Ryo Nakamura, Ikuko Nakamura, Hitoshi Nakashima, Mamoru Nanasato, Isao Nishi, Shinichi Niwano, Shuichi Nomura, Nozomu Oda, Shio Oguchi, Mitsutoshi Oguri, Arihide Okahara, Masaaki Okutsu, Fumitake Ozaki, Michishige Ozeki, Tomoko Saisu, Yuichi Saito, Makoto Saitoh, Yosuke Saka, Yoshihiko Sakai, Kazushi Sakane, Ikki Sakuma, Shakya Sandeep, Hiroaki Sano, Hisakuni Sekino, Yuka Senoo, Kensaku Shibata, Yoshisato Shibata, Takahisa Shibata, Akina Shiga, Kazuki Shiina, Michio Shimabukuro, Yusaku Shimbo, Wataru Shimizu, Masahisa Shimpo, Takeshi Soeki, Koichi Sohmiya, Hiroyuki Suzuki, Susumu Suzuki, Makoto Suzuki, Nobuhiro Tahara, Tazu Tahara, Sadako Takahashi, Bonpei Takase, Kaoru Takegami, Tomoko Takiguchi, Tomonobu Takikawa, Ai Tamura, Tomoaki Tanaka, Akihito Tanaka, Hiroyuki Tanaka, Jun Tanigawa, Daisuke Tanimura, Yosuke Tatami, Takashi Terano, Fumio Terasaki, Tomoyuki Tobushi, Seiko Tokoi, Toshiyuki Tsubouchi, Daigaku Uchida, Tomohiro Ueda, Rie Ueno, Hiromi Ueno, Chikara Ueyama, Tetsuzo Wakatsuki, Tomohiko Watanabe, Masato Watarai, Isao Yaguchi, Ayumu Yajima, Jiko Yamada, Kyohei Yamamoto, Sachiko Yamauchi, Yohei Yamauchi, Naoto Yokota, Tomohikov Yoshida, Goro Yoshioka, Junya Ako, Kazuo Kitagawa, Wataru Shimizu, Masaharu Ishihara, Tomoko Ishizu, Shinichiro Ueda, Atsushi Tanaka, Jun-ichi Oyama, Mikiko Kagiyama

**Affiliations:** 1 Department of Cardiovascular Medicine, Tokushima University Hospital, Tokushima, Japan; 2 Department of Medical Statistics, Osaka City University Graduate School of Medicine, Osaka, Japan; 3 Department of Cardiovascular Medicine, Saga University, Saga, Japan; 4 Department of Cardiovascular Medicine, JR Hiroshima Hospital, Hiroshima, Japan; 5 Department of Cardiovascular Medicine and Hypertension, Graduate School of Medical and Dental Sciences, Kagoshima University, Kagoshima, Japan; 6 Division of Cardiovascular Medicine, Department of Medicine, Kurume University School of Medicine, Kurume, Japan; 7 Department of General Internal Medicine, Saitama Red Cross Hospital, Saitama, Japan; 8 Department of Cardiology, Japanese Red Cross Aichi Medical Center Nagoya Daiichi Hospital, Nagoya, Japan; 9 Division of Cardiovascular Medicine, Department of Medicine, Jichi Medical University School of Medicine, Shimotsuke, Japan; 10 Department of Community Medicine for Cardiology, Tokushima University Graduate School of Biomedical Sciences, Tokushima, Japan; 11 Saga University, Saga, Japan; 12 Nagoya University Graduate School of Medicine, Nagoya, Japan; 13 Dokkyo Medical University, Mibu, Japan; 14 Tokushima University Graduate School, Tokushima, Japan; 15 Kagoshima University, Kagoshima, Japan; 16 Chiba University Graduate School of Medicine, Chiba, Japan; 17 Jichi Medical University School of Medicine, Shimotsuke, Japan; 18 Juntendo University Graduate School of Medicine, Tokyo, Japan; 19 Osaka University, Graduate School of Medicine, Suita, Japan; 20 Kurume University School of Medicine, Kurume, Japan; 21 Nagasaki University Graduate School of Bivomedical Sciences, Nagasaki, Japan; 22 University of the Ryukyus, Okinawa, Japan; 23 Kitasato University School of Medicine, Sagamihara, Japan; 24 Graduate School of Comprehensive Human Sciences, University of Tsukuba, Tsukuba, Japan; 25 Nagoya Daini Red Cross Hospital, Nagoya, Japan; 26 Kameda Medical Center, Komogawa, Japan; 27 Nippon Medical School, Tokyo, Japan; 28 Nagoya Ekisaikai Hospital, Nagoya, Japan; 29 Chiba Aoba Municipal Hospital, Chiba, Japan; 30 Isumi Medical Center, Isumi, Japan; 31 Japanese Red Crossv Nagoya Daini Hospital, Nagoya, Japan; 32 Hiroshima General Hospital of West Japan Railway Company, Hiroshima, Japan; 33 Dokkyo Medical University Saitama Medical Center, Koshigaya, Japan; 34 Graduate School of Medicine, Chiba University, Chiba, Japan; 35 Osaka Medical College, Takatsuki, Japan; 36 Tokushima University Graduate School of Biomedical Sciences, Tokushima, Japan; 37 Kimitsu Chuo Hospital, Kisarazu, Japan; 38 Miyazaki Medical Association Hospital, Miyazaki, Japan; 39 Kagoshima City Hospital, Kagoshima, Japan; 40 National Defense Medical College, Tokorozawa, Japan; 41 Sekino Hospital, Tokyo, Japan; 42 Sasebo City General Hospital, Sasebo, Japan; 43 University of the Ryuvkyus, Nishihara, Japan; 44 Hyogo College of Medicine, Nishinomiya, Japan; 45 Fukuoka Saiseikai Futsukaichi Hospital, Chikushino, Japan; 46 Japanese Red Cross Nagoya Daiichi Hospital, Nagoya, Japan; 47 Kasugai Municipal Hospital, Kasugai, Japan; 48 Tokyo Women’s Medical University, Tokyo, Japan; 49 Asahi General Hospital, Asahi, Japan; 50 Nagasaki University Graduate School of Biomedical Sciences, Nagoya, Japan; 51 Kobe City Medical Center General Hospital, Kobe, Japan; 52 Institute of Advanced Medical Sciences, Tokushima University, Tokushima, Japan; 53 The Institute for Adult Diseases, Asahi Life Foundation, Tokyo, Japan; 54 Graduate School of Biomedical and Health Sciences, Hiroshima University, Hiroshima, Japan; 55 Imari Arita Kyoritsu Hvospital, Matsuura, Japan; 56 Saga-Ken Medical Centre Koseikan, Saga, Japan; 57 Nozaki Tokushukai Hospital, Daito, Japan; 58 Ryukyu University Hospital, Nishihara, Japan; 59 National Hospital Organization Kagoshima Medical Center, Kagoshima, Japan; 60 National Hospital Organization Kasumigaura Medical Center, Tsuchiura, Japan; 61 Tokyo Medical University, Tokyo, Japan; 62 Chiba University Hospital, Chiba, Japan; 63 Nishio Municipal Hospital, Nishio, Japan; 64 Kameda Medical Center, Kamogawa, Japan; 65 Niko Clinic, Takeo, Japan; 66 Hotaruno Central Clinic, Kisarazu, Japan; 67 Gifu Prefectural Tajimi vHospital, Tajimi, Japan; 68 Anjo Kosei Hospital, Anjo, Japan; 69 Yokota Naika, Miyazaki, Japan; 70 Tsukuba Echo Core Laboratory. LLC, Tsukuba University, Tsukuba, Japan; 71 Clinical Research Management Center, University of the Ryukyus, Okinawa, Japan; 72 Clinical Research Support Center, University of the Ryukyus, Okinawa, Japan; 73 Nouvelle Place Inc, Tokyo, Japan; 74 Organization for Clinical Medicine Promotion, Tokyo, Japan

**Keywords:** febuxostat, hyperuricemia, echocardiography, diastolic function, NT-proBNP

## Abstract

Hyperuricemia is related to an increased risk of cardiovascular events from a meta-analysis and antihyperuricemia agents may influence to cardiac function. We evaluated the effect of febuxostat on echocardiographic parameters of diastolic function in patients with asymptomatic hyperuricemia as a prespecified endpoint in the subanalysis of the PRIZE study. Patients in the PRIZE study were assigned randomly to either add-on febuxostat treatment group or control group with only appropriate lifestyle modification. Of the 514 patients in the overall study, 65 patients (31 in the febuxostat group and 34 in the control group) who had complete follow-up echocardiographic data of the ratio of peak early diastolic transmitral flow velocity (E) to peak early diastolic mitral annular velocity (e′) at baseline and after 12 and 24 months were included. The primary endpoint was a comparison of the changes in the E/e′ between the two groups from baseline to 24 months. Interestingly, e′ was slightly decreased in the control group compared with in the febuxostat group (treatment *p* = 0.068, time, *p* = 0.337, treatment × Time, *p* = 0.217). As a result, there were significant increases in E/e′ (treatment *p* = 0.045, time, *p* = 0.177, treatment × time, *p* = 0.137) after 24 months in the control group compared with the febuxostat group. There was no significant difference in the serum levels of N-terminal-pro brain natriuretic peptide and high-sensitive troponin I between the two groups during the study period. In conclusions, additional febuxostat treatment in patients with asymptomatic hyperuricemia for 24 months might have a potential of preventable effects on the impaired diastolic dysfunction.

## Introduction

Hyperuricemia can be associated with cardiometabolic abnormalities and the association leads to the development of atherosclerosis and resultant cardiovascular disease [1–3]. Several studies investigated the association between hyperuricemia and echocardiographic variables [4–7]. Krishnan et al. showed the correlation between the left ventricular (LV) mass index and serum uric acid (SUA) level [8]. In addition, Lin et al. also showed the LV diastolic functional parameters were associated with gout [9]. The association between hyperuricemia and cardiac function is a key mechanism of hyperuricemia on cardiovascular diseases. Therefore, it is clinically required to evaluate the impact of antihyperuricemia agents on cardiac function from the randomized cohort study.

Febuxostat is a nonpurine selective inhibitor of xanthine oxidase for the treatment of hyperuricemia and gout. Febuxostat has greater potency for inhibition of xanthine oxidase activity and more urate-lowering efficacy than allopurinol. Accordingly, febuxostat may have superior antioxidative and antiatherogenic effects to allopurinol [10, 11]. According to the link between hyperuricemia and cardiac function, we hypothesized that the febuxostat can influence the LV diastolic function in patients with hyperuricemia. The PRIZE (program of vascular evaluation under uric acid control by xanthine oxidase inhibitor, febuxostat: multicenter, randomized controlled) study (University Hospital Medical Information Network Clinical Trial Registry UMIN000012911) was a prospective multicenter study conducted in Japanese patients with asymptomatic hyperuricemia to evaluate the inhibitory effect of febuxostat on the progression of atherosclerosis based on carotid-artery intima-media thickness assessed by ultrasonography over a 2-year follow-up period [12]. To elucidate the effect of febuxostat on echocardiographic parameters, we investigated the effect of the febuxostat on 2-dimensional and Doppler echocardiographic parameters and biomarkers from baseline to 24 months as a prespecified endpoint in the subanalysis of the PRIZE study (University Hospital Medical Information Network Clinical Trial Registry UMIN000041322).

### Study design

Details of the PRIZE study design and inclusion and exclusion criteria for the study have been published elsewhere [13]. In brief, the study was a multicenter, randomized, prospective, open-label, blinded-endpoint trial accomplished by 48 sites throughout Japan. A total of 514 adults (aged ≥20 years) who had hyperuricemia with SUA > 7.0 mg/dL enrolled in this study between May 2014 and August 2018. Key exclusion criteria were the administration of any SUA-lowering agents within the 8-week period prior to the assessment of eligibility, the presence of gouty tophus, or symptoms of gout arthritis within one year before assessment of eligibility. The patients were randomly assigned using a 1:1 ratio to either add-on febuxostat treatment (febuxostat group, *n* = 257) or an appropriate lifestyle modification for hyperuricemia, such as healthy diet and exercise therapy without febuxostat (control group, *n* = 257). The original primary endpoint of the PRIZE study was the change in mean common carotid IMT 24 months after treatment randomization. We prespecified the echocardiographic endpoint in the subanalysis of the PRIZE study. Echocardiography was performed as an ad hoc examination at baseline and 12 and 24 months after treatment randomization. The ethical committees of each participating institution approved the study protocol, with written informed consent for participation in the study being obtained from all subjects.

### Study population

**Fig. 1:**
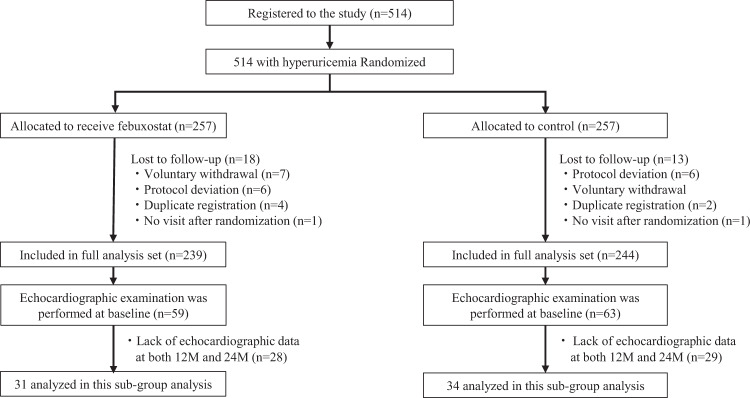
Patient selection

The echocardiographic examination was performed in 59 patients in the febuxostat group and in 63 patients in the control group at the baseline. We excluded the patients with the lack of echocardiographic data at both 12 and 24 months to analyze the changes of echocardiographic parameters during follow-up. After exclusions, 31 patients in the febuxostat group and in 34 patients in the control group were included for the final analysis (Fig. 1).

### Echocardiographic assessment

Echocardiography was performed by the commercially available ultrasound machine and various hemodynamic parameters were measured at each institution. The recordings and measurements were according to the guidelines issued by American Society of Echocardiography [14]. Left ventricular ejection fraction (LVEF) and left atrial volume were measured and calculated from the apical two- and four-chamber view using bi-plane disk methods. LV mass by M-mode echocardiography was derived using the American Society of Echocardiography’s guideline formula. Transmitral flow velocity was recorded from the apical long-axis or four-chamber view. The peak early diastolic (E) and the peak atrial systolic (A) velocities, and deceleration time of early TMF velocity were measured. The mitral annular motion velocity pattern was recorded from the apical four-chamber view with a sample volume placed at the lateral or septal side of the mitral annulus using pulsed tissue Doppler echocardiography. Early diastolic (e′) peak velocities were measured and the ratio of E to e′ (E/e′) was calculated using averaged values. This index was used to be a marker of LV filling pressure.

### Laboratory examination

Blood samples were collected at baseline and after 12 and 24 months. The serum levels of N-terminal pro-brain natriuretic peptide (NT-proBNP) and high-sensitive troponin I were measured in a centralized laboratory (SRL Co. Tokyo, Japan).

### Statistical analysis

For the baseline variables, the summary statistics were expressed as frequencies and proportions for categorical data and mean (SD) or median (interquartile range) for continuous variables. Mean percentage changes in the echocardiographic variables and laboratory parameters from baseline to 12 and 24 months and its 95% confidence interval (CI), estimated with the repeated measures regression model using the mixed-effect model. To assess change from the baseline in the model with the logarithmic the echocardiographic variables, the baseline logarithmic variable was further adjusted in the model. Comparisons of changes in the other efficacy endpoints between the treatment groups were performed by examining the interaction between the treatment groups and follow-up time, which statistical tests for differences in rates of change between the regression slopes of the weight over time between the febuxostat group and control group. All P values were two-sided with a level of significance of 0.05, and there were adjustments for baseline data. We were unable to adjust for the other variables in this analysis due to the limited number of patients. All statistical analyses were performed using R 4.0.1. (R Foundation for Statistical Computing, Vienna, Austria.)

## Results

### Baseline clinical characteristics

Comparisons of baseline clinical characteristics of the febuxostat group and control group were shown in Table 1. In the febuxostat group, 25.8% of the patients received 10 mg, 22.6% received 20 mg, and 48.4% received 40 mg daily as the final dose of febuxostat. While around 90% of the subjects had hypertension in the two groups, the blood pressure was well controlled in both groups. Dyslipidemia also existed in around 60% of the patients in each group; however, the average serum total cholesterol level was within the normal range. There was no significant difference in any examined variable between the two groups at baseline except the estimated glomerular filtration rate which was slightly higher in the control group than in the febuxostat group (*p* = 0.020). The uric acid level was significantly lower in the control group (*p* = 0.031). Table 2 showed the baseline echocardiographic variables in both groups. At baseline, averaged e′ in febuxostat group was lower and E/e’ in the febuxostat group was higher than in the control group. There was no significant difference in the other variables between the two groups. Around 70% patients had normal or concentric remodeling LV geometry.

**Table 1:** Clinical characteristics at baseline

	ALL	Control	Febuxostat	P value
Number	65	34	31	
Clinical background				
Age, year	71 [63,77]	71 [63,77]	71 [63,77]	1.000
Male, %	80 (52)	88 (30)	71 (22)	0.082
Body mass index	25.1 [22.2, 26.9]	25.6 [22.9, 27.4]	24.7 [22.1, 26.4]	0.482
Systolic BP, mmHg	128 [118, 133]	128 [120, 132]	126 [115, 133]	0.818
Diastolic BP, mmHg	72 [66,80]	77 [68,80]	72 [65,79]	0.252
Heart rate, bpm	62 [58,72]	63 [56,71]	62 [59,72]	0.555
Clinical history				
Smoker, %, (*n*)	55.4 (36)	64.7 (22)	45.2 (14)	0.113
Hypertension, %, (*n*)	90.8 (59)	88.2 (30)	93.5 (29)	0.460
Diabetes mellitus, %, (*n*)	41.5 (27)	38.2 (13)	45.2 (14)	0.571
Dyslipidemia, %, (*n*)	63.1 (41)	55.9 (19)	71.0 (22)	0.208
Myocardial infarction, %, (*n*)	13.8 (9)	8.8 (3)	19.4 (6)	0.220
PCI, %, (*n*)	24.6 (16)	17.6 (6)	32.3 (10)	0.172
CABG, %, (*n*)	6.2 (4)	5.9 (2)	6.5 (2)	0.924
Stroke, %, (*n*)	4.6 (3)	5.9 (2)	3.2 (1)	0.610
Heart failure, %, (*n*)	16.9 (11)	8.8 (3)	25.8 (8)	0.068
Medications				
ARB %, (*n*)	61.5 (40)	58.8 (20)	64.5 (20)	0.638
ACEI %, (*n*)	12.8 (8)	11.8 (4)	12.9 (4)	0.889
Beta blocker %, (*n*)	44.6 (29)	45.1 (15)	45.2 (14)	0.933
Diuretic %, (*n*)	33.8 (22)	32.4 (11)	35.5 (11)	0.790
Statin %, (*n*)	47.7 (31)	44.1 (15)	51.6 (16)	0.610
Anti-platelet %, (*n*)	47.7 (31)	50.0 (17)	45.2 (14)	0.696
Aspirin %, (*n*)	41.5 (27)	38.2 (13)	45.2 (14)	0.571
Laboratory Data				
Total cholesterol, mmol/L	4.56 [4.01, 5.25]	4.55 [4.16, 5.25]	4.58 [3.98, 5.31]	0.968
eGFR, mL/min/1.73m^2^	57.5 [47.0, 66.8]	60.1 [53.2, 68.9]	48.4 [42.5, 59.7]	0.020
Uric acid, mg/dL	7.65 [7.10, 8.28]	7.50 [7.10, 7.90]	8.10 [7.35, 8.70]	0.031
NT-proBNP, pg/mL	99.5 [33.3, 314.1]	98.7 [33.3, 222.4]	100.4 [37.3, 361.4]	0.624
Troponin I, pg/mL	5.15 [3.20, 9.70]	5.10 [3.30, 8.00]	5.30 [3.15, 10.55]	0.867

*BP* blood pressure, *PCI* percutaneous coronary intervention, *ACEi* angiotensin-converting-enzyme inhibitor, *ARB* angiotensin II receptor blocker, *eGFR* estimate glomerular filtration rate, *NT-proBNP* N-terminal-pro brain natriuretic peptide.

Data are presented as the number of patients (percentage), mean ± SD or median (interquartile range).

**Table 2:** Echocardiographic variables at baseline

	ALL	Control	Febuxostat	P value
Number	65	34	31	
Echocardiographic variables				
TMF-E	66 [57,80]	64 [57,80]	66 [58,81]	0.480
Averaged e’	7.65 [6.25, 9.10]	7.75 [6.95, 10.20]	6.09 [4.80, 7.99]	0.028
Averaged E/e’	8.04 [7.01, 10.01]	7.90 [6.32, 9.08]	9.18 [7.86, 11.67]	0.032
TMF-A	79 [67,91]	83 [69,93]	74 [66,89]	0.430
E/A	0.79 [0.74, 1.11]	0.77 [0.69, 1.13]	0.86 [0.76, 1.08]	0.302
Deceleration time	217 [192, 241]	220 [200, 247]	210 [186, 234]	0.349
LV end-diastolic dimension	48.0 [45.0, 52.0]	48.2 [44.9, 52.3]	47.0 [45.0, 52.0]	0.804
LV end-systolic dimension	30.1 [27.7, 34.1]	31.0 [27.7, 34.0]	30.0 [27.8, 36.3]	0.946
LV ejection fraction	63.9 [56.4, 69.0]	64.0 [58.5, 69.0]	63.0 [56.4, 69.0]	0.602
LV mass index	101 [88, 123]	102 [86, 121]	99 [91, 128]	0.611
Categories of LV hypertrophy				0.774
Normal	42.9%	47.6%	38.1%	
Concentric remodeling	31.0%	23.8%	38.1%	
Concentric hypertrophy	11.9%	14.3%	9.5%	
Eccentric hypertrophy	14.3%	14.3%	14.3%	
LA volume	58 [45,77]	59 [42,77]	54 [49,70]	0.923

*TMF-E* early diastolic transmitral flow wave, *e’* early mitral annular velocity, *TMF-A* late diastolic transmitral flow wave, *LV* left ventricular, *LA*, left atrial.

Data are presented as median (interquartile range).

### Laboratory data

**Fig. 2:**
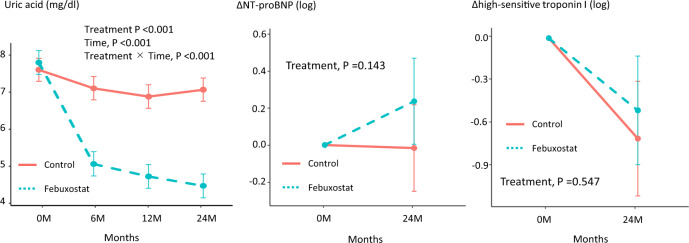
Changes in uric acid, NT-pro BNP, and high-sensitive troponin I at 12 and 24 months in the two treatment groups

Figure 2 showed the changes in laboratory data, including uric acid, NT-pro BNP, and high-sensitive troponin I. There were significant differences in the SUA levels between the treatment groups at 6, 12, and 24 months, and the final SUA levels were 4.46 and 7.07 mg/dL in the febuxostat and control groups, respectively (treatment, time and treatment × time, all *p* < 0.001). There were no significant differences in serum level of NT-pro BNP (treatment *p* = 0.1143) and high-sensitive troponin I (treatment *p* = 0.547) in both groups throughout this study.

### Echocardiographic parameters

**Fig. 3:**
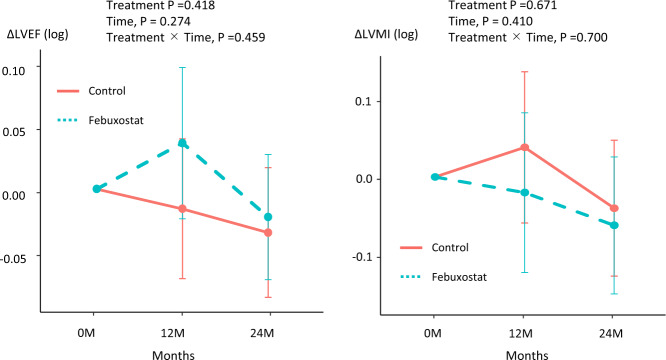
Changes in left ventricular ejection fraction (LVEF) and LV mass index (LVMI) at 12 and 24 months in the two treatment groups

**Fig. 4:**
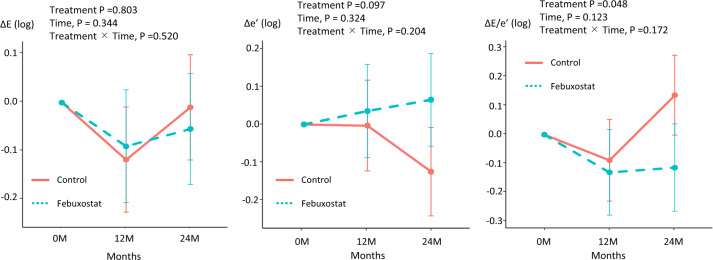
Changes in early diastolic transmitral flow velocity (E), early diastolic mitral annular velocity (e’), and E/e’ at 12 and 24 months in the two treatment groups

Comparisons of echocardiographic parameters at baseline and after 12 and 24 months of treatment in the febuxostat group and control group are shown in Figs. 3 and 4. There were no significant differences of LV ejection fraction (treatment *p* = 0.422, time, *p* = 0.283, treatment × Time, *p* = 0.469; Fig. 3) and LV mass index (treatment *p* = 0.713, time, *p* = 0.532, treatment × Time, *p* = 0.778; Fig. 3) at baseline and after 12 and 24 months in both groups. In the Doppler parameters, there were no significant difference of E wave (treatment *p* = 0.758, time, *p* *=* 0.325, treatment × Time, *p* *=* 0.486; Fig. 4) at baseline and after 12 and 24 months in both groups. Interestingly, e’ was slightly decreased in the control group compared with in the febuxostat group (treatment *p* = 0.068, time, *p* = 0.337, treatment × Time, *p* = 0.217; Fig. 4). As a result, there were significant difference between the control group and febuxostat group in E/e’ (treatment *p* = 0.045, time, *p* = 0.077, treatment × Time, *p* = 0.137; Fig. 4) after 24 months (control group 0.15 [0.001–0.291], febuxostat group -0.13 [-0.289–0.031], *p* = 0.010). We confirmed that there is no adverse effect of febuxostat on echocardiographic parameters and the febuxostat may lead to preventing the impaired diastolic dysfunction.

## Discussion

**Fig. 5:**
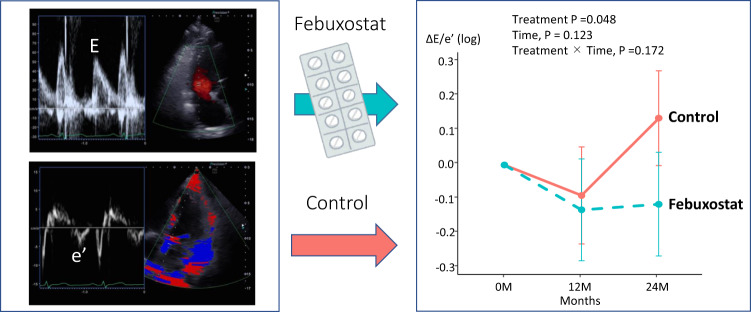
Graphical Abstract: This study was a post-hoc subgroup analysis of the PRIZE trial that focused on the effect of febuxostat on echocardiographic variables including diastolic functional parameters

This study was a post hoc subgroup analysis of the PRIZE trial that focused on the effect of febuxostat on echocardiographic variables, including diastolic functional parameters. The key finding of the study was that the addition of febuxostat significantly prevented the worsen diastolic function (e’ and E/e′) compared to control group (Fig. 5). However, there is no significant differences of other parameters including LV mass index and LVEF between the two groups. Moreover, there is no significant differences in the biomarkers including NT-proBNP and high-sensitive troponin I. According to these results, it seems that febuxostat therapy may have a protective effect on cardiac diastolic function independent from LV morphological changes or biochemical changes. However, the present study should be considered as a proof of concept, and we believe that larger prospective multicenter studies using the advanced diastolic functional markers (e.g., strain imaging) are warranted to confirm the cardiovascular protective effects on diastolic function by febuxostat.

### Impact of febuxostat on systolic function

Elevated SUA has been shown to inhibit nitric oxide production by vascular endothelial cells and also to inhibit their proliferation and migration [15]. In the clinical trial, there are several observational studies and meta-analyses that described elevations of SUA as an independent marker of poor cardiac function, mortality, and poor exercise capacity [16–18]. Increased SUA levels might lead to the echocardiographic abnormalities through impacts on endothelial function and inflammation. Thus, SUA may represent not only an actual prognostic marker of cardiovascular diseases but also a potential target for intervention to treat cardiac function. Several investigators have shown that LV systolic function, such as LVEF and LV mass, improved by treatment of elevated SUA [8, 19]. Our results did not show results consistent with these previous results for the positive effects on LV systolic function. Our data showed that there are no significant differences of LVEF and LV mass between the two groups. One possible explanation was that the baseline LVEF and LV mass were completely within the normal range, then no change occurred during follow-up. We need a prospective randomized study for advanced HF. Another difference between our study and previous studies was that our control subjects underwent only appropriate lifestyle modifications for hyperuricemia.

### Impact of febuxostat on diastolic function

In this study, additional treatment of febuxostat to usual hyperuricemia treatment significantly decreased the annual drop of e′ and elevation of E/e′, suggesting a preventive effect of febuxostat on LV compliance and diastolic dysfunction. The index of diastolic function is gradually decreased in aging. From the data of reference values, e′ decreases by 0.1 cm/sec per year [20]. In the control group, the e′ decreased from 8.5 to 7.7 after 2 years. Thus, there may be a decrease in diastolic function beyond age in patients with asymptomatic hyperuricemia.

Febuxostat treatment did not influence LV systolic functional parameters and cardiac structures or biomarkers, such as NT-proBNP. Some basic studies showed that treatment of SUA decreased myocardial indices of oxidative stress, as well as cardiac tissue xanthine oxidase highlights the potential direct antioxidant effects of the treatment of SUA compound as well as its uric acid and tissue xanthine oxidase lowering properties [21]. Additionally, our study population was consisted of 80% of all subjects in hypertension. Hyperuricemia is well known to be associated with LV hypertrophy and LV diastolic dysfunction in hypertensive patients [22, 23]. Hyperuricemia is associated with hypertensive heart disease, and hyperuricemia has an increased risk of coronary or cerebrovascular disease compared to hypertensive patients without hyperuricemia [24]. For these reasons, the protective effects on LV diastolic dysfunction have been detected in our cohort. However, in our data, the mechanisms for preventive effects of febuxostat on diastolic function were not fully explained. This study period may not have been long enough to assess the benefits of febuxostat. Moreover, the treatment effects of febuxostat might be stronger in patients with advanced HF than in patients with relatively normal cardiac function.

One possible mechanism is that the elevation in serum uric acid could be a reflection of increased xanthine oxidase activity, resulting in abnormal energy metabolism in cardiomyocytes [25]. This pathway might play an important role in diastolic dysfunction. Furthermore, increased activity of xanthine oxidase causes the release of free radicals and increased oxidative stress [26]. Oxidative stress lead to the pathogenesis of diastolic dysfunction. Because the precise mechanisms by which febuxostat treatment suppressed the increase in the diastolic parameter are not confirmed in our study, both basic and clinical data should be assessed in further studies.

Previous studies showed a J-curve association between uric acid and aortic diseases, including hypertension and low levels of SUA might be associated with increased cardiovascular diseases [27, 28]. In our cohort, febuxostat did not adversely affect the LV systolic and diastolic function in patients with asymptomatic hyperuricemia. Thus, in the range of SUA from our cohort, the J-curve association between uric acid and cardiac function was not detected, and lowering medication of hypouricemia should be used in hypouricemia for the cardiac function.

### Clinical implication

In this study, e′ decreased and E/e′ increased after 24 months in the control group, suggesting LV compliance has been impaired. In contrast, these parameters showed no significant changes in the febuxostat group. Thus, it is suggested that febuxostat at least did not adversely affect the LV compliance; possibly it acted rather protective for the impairment of LV compliance. We were unable to show the strong evidence of febuxostat to the LV diastolic parameters in the present study, it may be accomplished by long-term observation or using advanced HF cohort.

## Limitation

This study was a subanalysis of the PRIZE study and echocardiography was not performed in all subjects. Moreover, the echocardiographic parameters including tricuspid regurgitant jet, pulmonary vein flows, and global longitudinal strains were not performed to assess the diastolic function in detail. Therefore, the number of study subjects was relatively small and included only Japanese patients. Due to the small number of patients, it was statistically difficult to evaluate the relationship between doses and echocardiographic parameters. Further studies enrolling a large number of subjects are needed to confirm the long-term effect of febuxostat on LV diastolic function in patients with hyperuricemia and advanced HF.

## Conclusion

Febuxostat might have a potential of preventable effects on the impaired diastolic dysfunction assessed by echocardiography.

## Data Availability

The datasets analyzed during the current study are available from the corresponding authors on reasonable request (contact via prizesub-secre@clin-med.org).
